# Effects of Different Motion Parameters on the Interaction of Fish School Subsystems

**DOI:** 10.3390/biomimetics8070510

**Published:** 2023-10-26

**Authors:** Feihu Zhang, Jianhua Pang, Zongduo Wu, Junkai Liu, Yifei Zhong

**Affiliations:** 1 School of Mechanical Engineering, Guangdong Ocean University, No. 1 Haida Road, Mazhang District, Zhanjiang 524088, China; 2 Shenzhen Institute of Guangdong Ocean University, No. 3 Binhai 2nd Road, Dapeng New District, Shenzhen 518120, China; 3 Guangdong Provincial Key Laboratory of Intelligent Equipment for South China Sea Marine Ranching, Guangdong Ocean University, No. 5, Haibin Avenue Middle, Development District, Zhanjiang 524088, China

**Keywords:** immersion boundary method, fish school, lattice Boltzmann method, biological movement

## Abstract

For a long time, fish school swimming has attracted a great deal of attention in biological systems, as fish schools can have complex hydrodynamic effects on individuals. This work adopted a non-iterative, immersed boundary–lattice Boltzmann method (IB–LBM). A numerical simulation of two-dimensional three-degree-of-freedom self-propelled fish, in side-by-side, staggered, and triangle formations, was conducted by adjusting spacing and motion parameters. A comprehensive analysis of individual speed gains and energy efficiencies in these formations was carried out. Furthermore, an analysis of the hydrodynamic characteristics of fish schools was performed, using instantaneous vorticity profiles and pressure fields. Certain studies have shown that passive interactions between individuals cannot always bring hydrodynamic benefits. The swimming efficiency of side-by-side formations in the same phase gradually increases as the distance decreases, but it also brings certain burdens to individuals when the phases are different. This paper also shows that the roles of passive interactions, spacing, and deflections affect fish school subsystems differently. When the low-pressure areas created by a wake vortex act on one side of an individual’s body, the tail-end fish are good at gaining hydrodynamic benefits from it. This effect is not universal, and the degree to which individuals benefit from changes in exercise parameters varies. This study provides a theoretical basis for bioinspired robots, as well as providing certain insights into the mechanism of collective biological movement.

## 1. Introduction

In biology, fish come together out of social relationships, and they usually swim in organized groups. Schools of fish swim for various reasons, such as finding prey, breeding, and avoiding predators [1,2]. In addition, schools of fish swimming together has been of interest for many researchers in fluid mechanics. School swimming is thought to help reduce metabolic rate and energy expenditure [3,4]. Multiple previous studies have obtained—by observing and measuring individual oxygen consumption, tail frequency, and kinematic parameters—experimental results that have confirmed the advantages of fish swimming in terms of motion hydrodynamics [5,6]. Furthermore, hydrodynamic interactions between individuals in a fish school are sufficient in producing coherent collective motion, thereby increasing speed and saving energy. In addition, such interactions characterize the role of fluids in groups [7].

Previous research has revealed the hydrodynamic advantages brought about by the high-density aggregation of fish. The wake generated by fish schools, among individuals, brings about improvements in hydrodynamic efficiency and thrust [8]. At the same time, the hydrodynamic effects caused by the high-density accumulation of fish schools are affected by the different composition patterns of fish schools. There are various composition patterns of fish swimming, which include series formations, side-by-side formations, staggered formations, and triangular formations [6,9]. Fish always hope to reap the hydrodynamic benefits of formations, and this is achieved by using their bodies to extract energy from the vortices created by the formations [10,11]. In addition to the hydrodynamic benefits obtained by individuals from the vortex, the formation density brought about by the spacing between individuals and changing the tail beat phase are also ways in which to improve hydrodynamic efficiency [12,13].

Several previous studies have shown that vortices in hydrodynamics provide benefits to fish during swimming. Vortices come from the rotation of the fluid masses generated by fluid passing through obstacles in nature, which produce dynamic effects by acting on or close to fish bodies. Liao et al. used motion data measured from live fish behind a D-shaped cylinder to find that trout changed their body motion, under the action of the vortex generated by the D-shaped cylinder, to obtain energy [10]. Based on the experimental observation results of Liao et al., Zhu et al. used numerical simulation combined with deep reinforcement learning (DRL) to adopt an adaptive control method for fish. The fish achieved labor savings from hydrodynamics by adjusting their body postures between the vortices [14].

At the same time, the swing of the fish’s tail fin will also generate vortices, which are similar to water jets that are ejected at high speed, thereby providing propulsion power for the fish’s movement. In fish schools, followers are often affected by the vortex generated by the leader. The existence of this vortex also reacts on the leader and produces hydrodynamic effects. Weihs proposed the vortex hypothesis and the channel effect. The “vortex hypothesis” postulates that the vortices generated by fish schools will have a positive impact on individuals. The “channel effect” refers to the different spacing between individuals in a school of fish, which bring about different hydrodynamic properties. These hypotheses are based on the non-viscosity theory, which posits that the tail-end fish improve their energy saving efficiency due to the vortex that is generated at the fronts of the fish; in addition, the propulsion force required for the fish school to form a diamond-shaped formation, due to the vortex effect, is smaller [8]. Based on the vortex hypothesis proposed above, Arranz et al. simulated a side-by-side formation of three-dimensional flexible plates to study the hydrodynamic influence of vortices on this collective effect. Given the different phase differences and vertical spacing between individuals, the results showed that the performance of the follower is affected by the vortices and vertical jets generated by the leader, as well as by the wake that is generated by itself [15]. Yu et al. used a deep reinforcement learning strategy to set the target reward value for the tail-end fish to follow the leader fish. By allowing individuals to independently choose the fish school structure, it was found that two self-propelled fish autonomously formed a side-by-side formation under the action of the vortex, and the average propulsion efficiency of the individuals in the fish school thus increased by 14% [16]. The above studies showed that the vortex hypothesis is supported.

The channel effect between individuals in fish schools has also been supported in previous studies. Channeling effects are often inseparable from the distance between individuals. The research of Li et al. showed that different formations are affected by spacing differently, due to the influence of the channel effect. For a series formation, a small spacing between fish is the most efficient, and for a rectangular formation, a large spacing is more suitable and produces the highest efficiency [17]. The combination of the vortex hypothesis and channel effect has a positive impact on fish schools. Pan et al. improved a fish leader’s thrust by 56% by changing the phase of a subsystem that was composed of fish school followers; furthermore, the follower’s efficiency and thrust increased by 58% and 108%, respectively [18]. Oza et al. showed that achieving a high thrust and obtaining high energy efficiency derive from different formation structures. Diamond formations provided the greatest hydrodynamic benefits to fish, compared to side-by-side and rectangular formations. Oza et al. differ from Weihs in that they showed that, in side-by-side formations, the direct effect of a vortex on the follower is not necessarily destructive [19].

The mechanism of propulsion performance was found to derive from flexible bodies and flapping wings in previous studies [20,21,22]. Researchers often restrict the swimmers’ degrees of freedom (DOF) or their given known path planning; however, this type of interference is not consistent with the fishes’ actual situation. Researchers have analyzed the movement of flexible bodies and the influence of fluid dynamics by using the movement of flexible bodies behind cylinders [23,24]. A recent study used a 1-DOF swimmer, and only gave the swimmers a longitudinal propulsion. Stable matrix and diamond formations can be formed through passive interaction [25]. Lateral and rotationally induced fluid–body interactions are often overlooked. Although certain studies have adopted 2-DOF swimmers, that is, endowing swimmers with longitudinal and lateral degrees of freedom, linear formations and diamond formations are often not stably maintained due to the lateral authority allowed; only specific formations such as side-by-side formations and staggered formations have been found to be stable [26]. To summarize, existing studies have usually limited the degrees of freedom of fish to maintain a specific formation and spacing; as such, they have often ignored the hydrodynamic effects of fluid feedback which change the positional relationship between fish individuals.

Therefore, the purpose of this study is to investigate the passive hydrodynamic effects of 3-DOF self-propelled swimmers in side-by-side formations, staggered formations, and triangular formations. By giving the self-propelled swimmers three degrees of freedom, the hydrodynamic impact of passive changes in the positions of the fish school, which are caused by the different motion parameters of the swimmer, is analyzed. Given the different initial spacings between individuals in a fish shoal, the hydrodynamic effects of spacing on fish shoals are explored, so as to reflect on the characteristics of hydrodynamic interactions. Changes are enacted in the phase difference and body fluctuation amplitude between individuals, and the performance improvements brought by passive changes in individual positions to fish, such as efficiency and speed, are explored. Such an approach is expected to further reveal the mechanism of the impact of changes in movement parameters on the hydrodynamic performance of fish schools.

The remainder of this paper is organized as follows. Section 2 presents the numerical model—a non-iterative, immersed boundary–lattice Boltzmann method (IB–LBM). The numerical simulation results will be discussed in Section 3. Section 4 gives the conclusion of this paper.

## 2. Methods

### 2.1. Fluid Systems—The Lattice Boltzmann Method

IB–LBM is used to solve the fluid–structure interaction system, in which the LBM acts on the solution of fluid. In recent years, LBM has been used by many researchers as an incompressible fluid solver; moreover, it has been developed rapidly and is currently indispensable [27,28]. The multiple-relaxation-time lattice Boltzmann equation is used in this work as follows:

(1) $$f_{i}\left(x+e_{i}\Delta t,t+\Delta t\right)-f_{i}\left(x,t\right)=\Omega_{i}\left(f\right),\;i=0,1\ldots 8$$

where *f* is the distribution function, ***x*** is the spatial coordinate, ***e_i_*** is the discrete velocity direction, *t* represents the time step, and $\Omega_{i}$ is the collision operator.

(2) $$\Omega_{i}=-{\left(M^{-1}SM\right)}_{ij}\left(f_{i}-f_{i}^{eq}\right),$$

where ***M*** is the 9×9 transformation matrix, ***S*** is the relaxation matrix [29,30], and $f_{i}^{eq}$ is the equilibrium distribution function.

(3) $$f_{i}^{eq}=w_{i}\rho \left[1+\frac{u\cdot e_{i}}{c_{s}^{2}}+\frac{{\left(u\cdot e_{i}\right)}^{2}}{2c_{s}^{4}}-\frac{u^{2}}{2c_{s}^{2}}\right],$$

where $w_{i}$ is the weight coefficient, ρ is the fluid density, u is the fluid velocity, $c_{s}$ is the sound velocity of the lattice, and $c_{s}=∆x/(∆t\sqrt{3})$. In this work, the D2Q9 model is used to study the two-dimensional fluid solver, where $e_{i}$ are (0, 0), (±1, 0), (0, ±1), and (±1, ±1).

(4) $$w_{i}=\left\{\begin{array}{c} 4/9,\;\;\;\;\;i=0,\\ 1/9,\;\;\;\;\;i=1,2,3,4\\ 1/36,\;\;\;\;i=5,6,7,8 \end{array}\right.$$

The macroscopic physical quantities of fluid density and velocity are obtained by the distribution function of each time step, and the pressure is related to fluid density and lattice sound velocity.

(5) $$\rho =\sum f_{i},\;p=\rho c_{s}^{2},\;\rho u=\sum {e_{i}f}_{i}.$$

### 2.2. Non-Iterative IBM of the Fluid Interactions with Fish Bodies

In this paper, the non-iterative IBM method is used to deal with the boundary of the interaction between fluids and fish bodies. This method does not need to add a force term to the LBM governing equation. Furthermore, the fluid and solid are in discrete states and belong to the Euler and Lagrange systems, respectively. This method uses an interpolation function to exchange information. Firstly, the interpolation function (Equation (6)) is used to obtain the possible distribution function $f_{i}^{*}(X)$ on the boundary points:

(6) $$f_{i}^{*}\left(X\right)=\sum_{x}f_{i}(x)\delta \left(x-X\right)(h{)}^{2},$$

where $\delta \left(r_{x},r_{y}\right)$ is the Dirac interpolation function and *h* is the grid spacing.

(7) $$\delta \left(r_{x},r_{y}\right)=\frac{1}{h^{2}}\xi \left(\frac{r_{x}}{h}\right)\xi \left(\frac{r_{y}}{h}\right),$$

(8) $$\xi (r)=\left\{\begin{array}{c} \frac{1}{8}\left(3-2|r|+\sqrt{1+4|r|-4r^{2}}\right),\;\;\;\;0\leq |r|<1,\\ \frac{1}{8}\left(5-2\left|r\right|-\sqrt{-7+12\left|r\right|-4r^{2}}\right),\;\;1\leq |r|<2,\\ 0\;\;\;\;\;\;\;\;\;\;\;\;\;\;\;\;\;\;\;\;|r|\geq 2. \end{array}\right.$$

The bounce-back rule is used to satisfy the non-slip distribution function at the boundary $f_{i}(X)$:

(9) $$f_{i}\left(X\right)=f_{\bar{i}}^{*}\left(X\right)+2\omega_{i}\rho \frac{e_{i}\cdot u_{b}}{c_{s}^{2}},$$

where $u_{b}$ is the boundary velocity and $f_{i}(X)$ is the expected distribution function at the boundary points. By introducing a parameter λ to adjust the deviation Equation (11), the non-slip boundary conditions can be precisely applied (this is the method proposed by Tao et al. [31]). A recent study by Tao et al. proposed a low computational load version [32].

(10) $$\Delta f_{i}\left(x\right)=\sum_{X}\lambda \left(n\right)\left(f_{i}\left(X\right)-f_{i}^{*}\left(X\right)\right)\delta \left(x-X\right)ds.$$

The distribution function after adjustment was assigned to the flow field, where ds is the arc length of the boundary, and the new distribution function in the flow field is $f_{i}\left(x\right)+\Delta f_{i}\left(x\right)$. This was performed to update the macroscopic flow field information, the macroscopic fluid density, and velocity, etc.

(11) $$\lambda \left(n\right)=\frac{1}{2\rho ds(h{)}^{2}\sum_{x}\sum_{X}\delta \left(x-X\right)\delta \left(x-X\right)},$$

Equation (12) was used to calculate the force exerted on the solid boundary from the flow field, and Equation (13) was used to calculate the total rotational torque.

(12) $$F_{s}=-\sum_{n}\sum_{i}e_{i}\left(\lambda \left(n\right)\left(f_{i}\left(X_{n}\right)-f_{i}^{*}\left(X_{n}\right)\right)\right)ds,$$

(13) $$T_{s}={-F}_{s}\times \left(X-r_{c}\right).$$

The translational and rotational motion parameters of each time step were solved by the display coupling method for convection–solid-coupled systems. This method is more effective and saves calculation time because it does not require iterative solutions [33,34].

(14) $$\frac{r_{c}^{i+1}-r_{c}^{i}}{\Delta t}=\frac{u_{c}^{i+1}+u_{c}^{i}}{2},$$

(15) $$m\frac{u_{c}^{i+1}-u_{c}^{i}}{\Delta t}=F_{s}^{i},$$

(16) $$\frac{\theta^{i+1}-\theta^{i}}{\Delta t}=\frac{{\phi_{s}}^{i+1}+{\phi_{s}}^{i}}{2},$$

(17) $$\frac{I_{s}^{i+1}+I_{s}^{i}}{2}\frac{{\phi_{s}}^{i+1}-{\phi_{s}}^{i}}{\Delta t}+{\phi_{s}}^{i+1}\frac{I_{s}^{i+1}-I_{s}^{i}}{\Delta t}=T_{s}^{i},$$

where $u_{c}$ is the center of mass speed, $\phi_{s}$ is the angular speed, and $I_{s}$ is the moment of inertia.

### 2.3. Configuration Scheme

**Figure 1:**
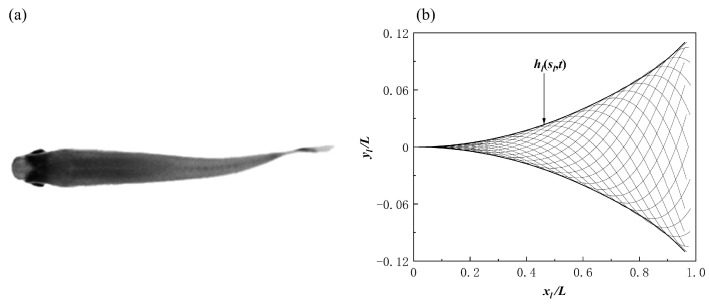
(**a**) The top view of red-nosed fish. (**b**) The lateral body displacement caused by the function of Equation (18).

**Figure 2:**
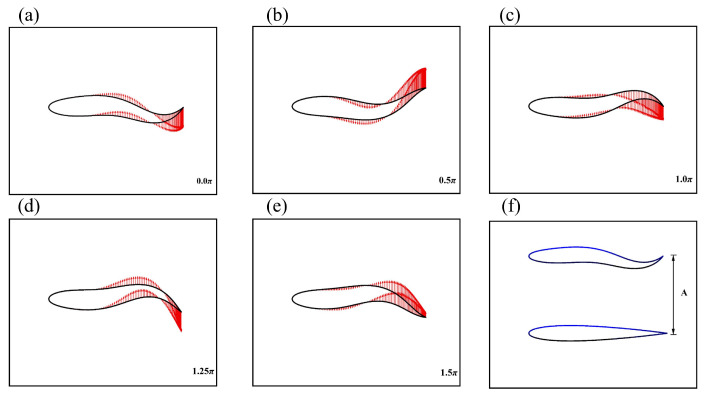
(**a**–**e**) Velocity vector diagrams of the fish silhouettes in different phases. (**f**) Schematic diagram of fish body tail amplitude.

**Figure 3:**
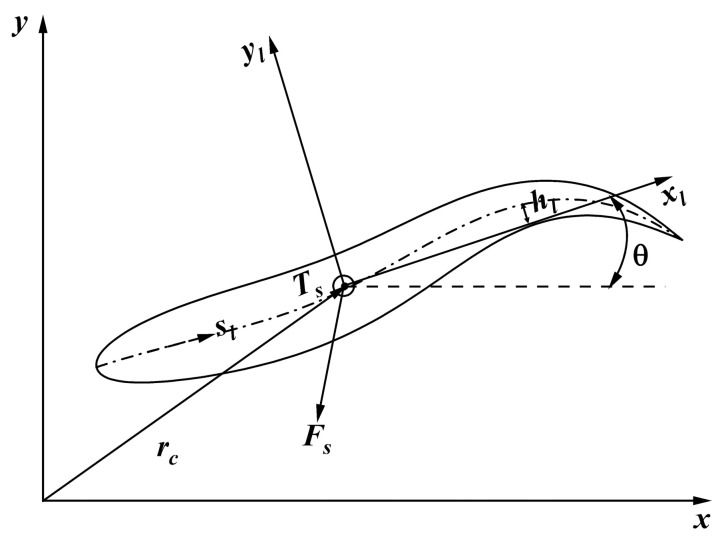
Schematic diagram of a 3-degree-of-freedom (3-DOF) swimmer model in two dimensions.

This section first discusses the formation design of fish schools and the selection of movement patterns with respect to individual fish. When studying the entire fish formation setup, three main factors were chiefly considered: the formation design of the fish school, the movement pattern of the fish bodies, and the initial spacing between the fish bodies. The main research purpose of this work is the impact of different movement patterns of individual fish on the fish school. The fish bodies were mainly arranged in three different formations: side-by-side, staggered, and triangle. The different movement patterns were mainly composed of different phase differences between the follower fish and the leader fish, as well as between different tail amplitudes. The overall formation design of the fish school and the different movement parameters and initial spacing of the follower fish are shown in Table 1. Certain studies have reported the effect of phase differences on the population [35]. The phase difference selection range of φ=0.0π−1.9π was given, and it was found that the follower efficiency was at its maximum when φ=1.6π. In this paper, the value of φ was selected within this range. In the experimental research report of [36], it was found that the influence of different incoming flow speeds on fish body movement mode selections and individual spacings was as follows: the horizontal spacing of fish was 0<Gx<0.3 L, the longitudinal spacing was 0.4 L<Gy<0.8 L, and the motion mode of the fish were the in-phase and anti-phase variants. This phenomenon can be explained by the overall efficiency of fish schools. In addition, it was found that the longitudinal and horizontal spacing between the fish individuals also affected them. The effect of high-density fish on leader and follower fishes in dense schools (0.4 *L*) and sparse schools (2.0 *L*) was studied. Following that, it was shown that the presence of follower fish led to a stronger rear pressure zone and a stronger hydrodynamic performance, with respect to the leader fish [37]. Certain other studies [38] have discussed the impact of different initial spacings and different tail amplitudes on fish schools in series configurations. In this study, the motion model of red-nosed four-tailed fish was selected, which is a widely used model for studying the influence of incoming flow on fish bodies, as shown in Figure 1a [36,39]. The wave equation describing the transverse displacement of the fish center line is as follows:

(18) $$h_{l}\left(s_{l},t\right)=A{\left(\frac{s_{l}}{L}\right)}^{2}\sin\left(2\pi \left(\frac{s_{l}}{\lambda }-\frac{t}{T}\right)+\varphi \right),$$

where $s_{l}$ is the horizontal coordinate of the center line of the fish bodies, *L* is the arc length of the center line of the fish, *t* is the time step, *A* is the amplitude of the fluctuation, λ is the wavelength, *T* is the wave period, and φ is the initial phase. The arc length of the center line of the fish bodies was constant at all time steps, and the transverse displacement of the center line at different time steps is shown in Figure 1b. This work used NACA0012 foil as the fish body model to study. The NACA0012 foil model describes the curved shape of a fish body. Half the thickness of the body is approximated by a fourth order polynomial:

(19) $$\frac{d_{l}}{L}=0.2610\sqrt{\frac{s_{l}}{L}}-0.3112\left(\frac{s_{l}}{L}\right)+0.1371{\left(\frac{s_{l}}{L}\right)}^{2}-0.0791{\left(\frac{s_{l}}{L}\right)}^{3}-0.0078{\left(\frac{s_{l}}{L}\right)}^{4},$$

where $d_{l}$ is half the thickness of the body and *L* is the length of the midline. The different phases of the fish body velocity vector diagram are shown in Figure 2a–e. The schematic diagram of the tail amplitude is shown in Figure 2f, and the selection of the tail amplitude of the follower fish in the whole fish school system is given in Table 1. A two-dimensional 3-DOF free swimmer model was adopted in this work, as shown in Figure 3. Body movement was understood as consisting of three parts: the body wave ($h_{l}$) in the local coordinate system, the translation of the bodies’ center of mass ($r_{c}$), and the rotation of the body about the center of mass (θ) in the global coordinate system. These categories can be expressed as follows:

(20) $$m\frac{d^{2}r_{c}}{dt^{2}}=F_{s},$$

(21) $$\frac{d}{dt}\left(I_{s}\frac{d\theta }{dt}\right)=T_{s},$$

where *m* is the mass of the fish bodies, $F_{s}$ is the total hydrodynamic force of the fish bodies, and $T_{s}$ is the total torque of the fish bodies rotating around the center of mass.

### 2.4. Performance Parameters

The swimming of the fish is unsteady, and the force that pushes the body forward changes with time; as such, the physical performance index of the fish also changes with time. As we are using the cost of transportation (*CoT*) to represent the self-propelled fish body energy efficiency [40,41,42], the definition of the transportation cost (*CoT*) expression can be shown as follows:

(22) $$P=\sum_{n}{-F}_{s}\left(X_{n}\right)\cdot u_{b}\left(X_{n}\right)$$

(23) $$CoT=\frac{\bar{P}}{m\bar{u}}$$

where P is the total input power, $F_{s}$ is the power of the boundary point, and $u_{b}$ is the local velocity of the boundary point. The cost of transportation (*CoT*) metric has the advantage of avoiding the inability to evaluate the efficiency of a self-propelled body when the average thrust of the self-propelled body is close to zero when cruising. The dimensionless expression for the cost of transportation (*CoT*) used in this work was formulated as follows:

(24) $$CoT=\frac{\bar{P}}{0.5\rho L^{3}/T^{2}\bar{u}}$$

## 3. Results

### 3.1. Verification of the Fluid–Structure Coupling System

In this section, the three numerical simulation cases and the grid-independent verification, which were designed to verify the reliability of the fluid–solid coupling solution system, are discussed. For these cases, an NVIDIA GeForce RTX 3060(USA) graphics cards and the CUDA C programming language, for the purpose of parallel computing to accelerate the flow field solving process, were used.

#### 3.1.1. Cylinder Fixed in a Uniform Incoming Flow

In this case, the values of *Re* = 40, *Re* = 100, and *Re* = 200 were designed to flow through a fixed cylinder. The Reynolds number was defined as $Re=\rho U_{0}D/\mu$, where ρ is the characteristic fluid density, $U_{0}$ is the far-field fluid velocity, D is the cylinder diameter, and μ is the fluid viscosity. The cylinder diameter was defined as *D* = 50 for grid spacing, and the computing domain size was 30D×30D. The average drag coefficient $C_{D}$ and the lift coefficient $∆C_{L}$ between peaks were calculated for comparison, as shown in Table 2. The drag coefficient $C_{D}$ and the dimensionless lift coefficient $C_{L}$ in the dimensionless form are shown in Equation (25), and the Strouhal number ***St*** is defined as follows:

(25) $$C_{D}\;=\frac{F_{D}}{0.5\rho U_{0}^{2}D},\;C_{L}=\frac{F_{L}}{0.5\rho U_{0}^{2}D},\;St=\frac{fD}{U_{0}}$$

**Figure 4:**
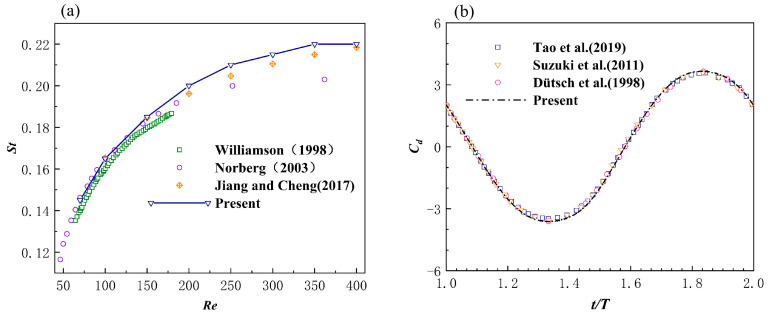
(**a**) Strouhal number as a function of the Reynolds number for a stationary cylinder [43,44,45]. (**b**) Comparison of the results of the drag coefficients of the oscillating cylinders [30,50,51].

By changing the Reynolds number from 70 to 400, the vortex shedding frequency of the Kármán vortex street was calculated so as to determine the Strouhalnumber St under different Reynolds numbers; the results of this can be observed in Figure 4a, and they can also be compared with Refs. [43,44,45]. The results show that the accuracy of the solver was guaranteed, and that $C_{D}$ and $∆C_{L}$ were in agreement with certain previously reported results [46,47,48,49].

**Table 2:** Comparison results of the average drag coefficient and peak-to-peak drag coefficient static cylinder at Re = 40, Re = 100, and Re = 200.

*Re*	40		100		200	
	${\overset{-}{C}}_{D}$	Δ*C_L_*	${\overset{-}{C}}_{D}$	Δ*C_L_*	${\overset{-}{C}}_{D}$	Δ*C_L_*
Present	1.582	-	1.345	0.67	1.339	1.41
Linnick [46]	1.61	-	1.38	0.674	1.37	1.40
Liu et al. [47]	-	-	1.35	0.678	1.31	1.38
Rosis [48]	1.50–1.71	-	1.28–1.46	0.56–0.78	1.31–1.45	1.20–1.56
Russell [49]	1.60	-	1.43	0.644	1.45	1.26

#### 3.1.2. Simulating a Cylinder Oscillating in a Stationary Fluid

In this case, to verify that the solver can simulate the motion of a rigid object in a fluid, a cylinder with an oscillating cylinder was selected to be placed in a static fluid. To prevent the boundary from affecting the calculation result, the size of the calculation domain was set at 30D×30D, where *D* represents the diameter of the cylinder. The horizontal oscillating motion of the cylinder was controlled by the function $X\left(t\right)=A_{max}sin(2\pi t/T)$, where $A_{max}$ is the amplitude of the oscillating motion and *T* is the period of the oscillation. The position of the center at time zero in the global coordinate system was $\left(x_{0},y_{0})=(15D,\;15D\right)$. The Reynolds number and Keulegan–Carpenter number are defined as $Re=\rho U_{max}D/\mu$ and $KC=U_{max}T/D$. Their values are 100 and 5, respectively, where $U_{max}$ is the maximum velocity of the cylinder.

**Figure 5:**
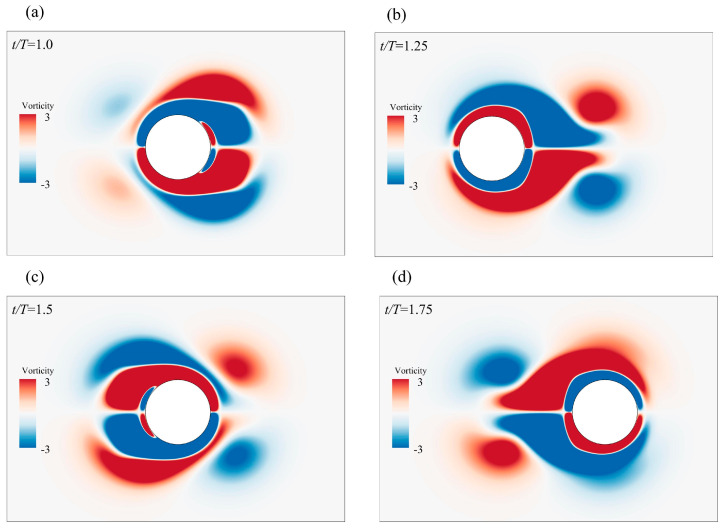
Instantaneous vorticity profile of an oscillating cylinder: (**a**) *t/T* = 1.0; (**b**) *t/T* = 1.25; (**c**) *t/T* = 1.5; and (**d**) *t/T* = 1.75.

The results of the drag coefficient calculated by the solver are shown in Figure 4b, and its results are in good agreement with certain previous reports [31,50,51]. The vortex generation process of the oscillating cylinder that was reproduced is shown in Figure 5, whereby the vorticity cloud diagrams were set at t/T=1.0, t/T=1.25, t/T=1.5, and t/T=1.75,respectively. The results were found to be consistent with the results of Liao et al. [52].

#### 3.1.3. Simulation of the Autonomous Propulsion of Anguilliform Swimmers in Stationary Fluids

**Figure 6:**
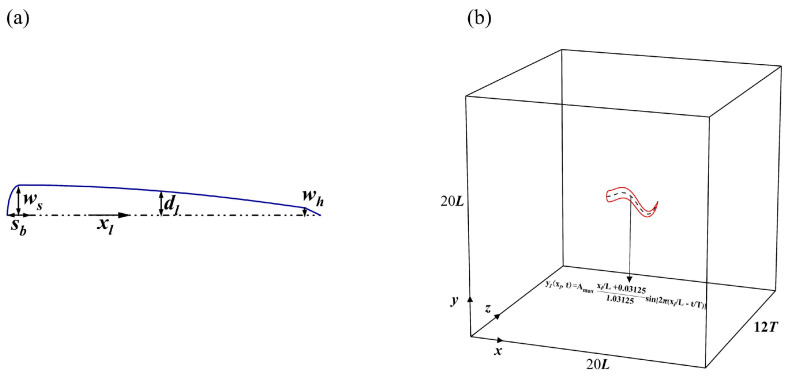
(**a**) Half the thickness of the body of an anguilliform swimmer. (**b**) The computational domain and total time steps for the anguilliform swimmer swimming validation.

In this case, the main purpose was to validate the ability of the current fluid solvers to simulate autonomously propelled swimmers. As such, an anguilliform swimmer for was selected for the verification of propulsion speed, where half of its body thickness is expressed as follows:

(26) $$d_{l}(x_{l})=\left\{\begin{array}{c} \sqrt{2w_{s}x_{l}-x_{l}^{2}},\;0\leq x_{l}<s_{b},\\ w_{s}-\left(w_{s}-w_{h}\right){\left(\frac{x_{l}-s_{t}}{s_{t}-s_{b}}\right)}^{2},s_{b}\leq x_{l}<s_{t},\\ w_{h}\frac{L-x_{l}}{L-s_{t}},\;s_{t}\leq x_{l}\leq L, \end{array}\right.$$

where $w_{s}=s_{b}=0.04\;L,s_{t}=0.95\;L$, and $w_{h}=0.01\;L$, as shown in Figure 6a. Figure 6b shows the autonomous propulsion verification problem of anguilliform swimmer swimming. Here, the computational domain size was set at 20 L×20 L, and the total time step was 12T. The wavy deformation of the anguilliform swimmer body is defined by the following formula:

(27) $$y_{l}\left(x_{l},t\right)=A_{max}\frac{x_{l}/L+0.03125}{1.03125}\sin\left[2\pi \left(\frac{x_{l}}{L}-\frac{t}{T}\right)\right]$$

where $A_{max}$ is the amplitude of the wave-like deformation of the swimmer’s body, L is the body length, $x_{l}$ is the longitudinal coordinate of the body midline, and T is the body wave period. Here, the Reynolds number is defined as $Re=\rho L^{2}/\mu$, and the body fluctuation period is T=1000Δt. In order to compare our results with the literature [53,54], the fluctuation amplitude was set as $A_{max}=0.125\;L$, and the Reynolds number was set as Re=7142.

**Figure 7:**
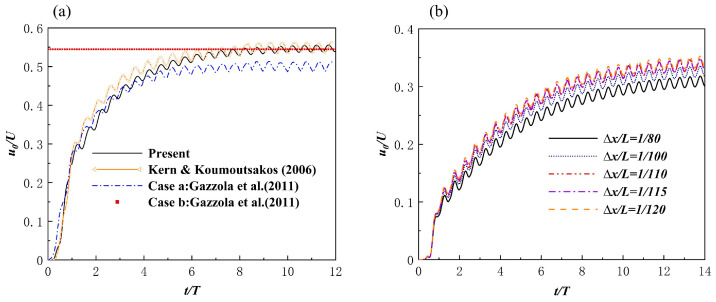
(**a**) Time history of the forward swimming speed of anguilliform swimmers compared with the results from other studies [53,54]. (**b**) Propelling speed of the red−nosed fish model at different grid resolutions.

Figure 7a shows the result of the solver calculating the forward propulsion velocity $u_{0}/U(U=L/T)$ of the anguilliform swimmer. When the anguilliform swimmer reached cruising speed, the model produced results that were particularly consistent with the results of Kern and Koumoutsakos [53] and Case b of Gazzola et al. [54], though they were slightly larger than the results of Case a of Gazzola et al. [54]. Figure 7b shows the forward speed of the red-nosed fish motion model (Equation (18)) at different grid resolutions. The Reynolds number was set as Re=1000. The motion parameters were A=0.11 L, λ=1.0 L, and T=1000Δt. The results showed that the convergence state was reached when the grid resolution was Δx/L=1/110.

### 3.2. Simulation Results

**Figure 8:**
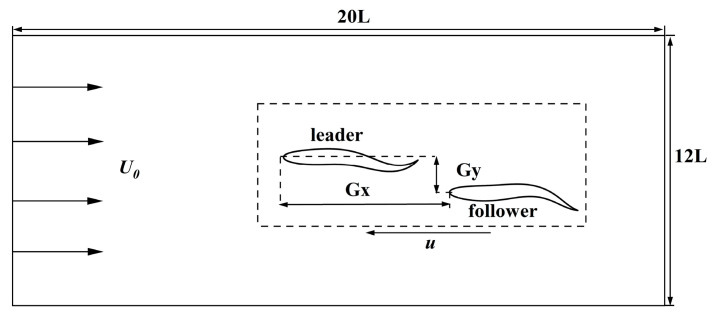
Schematic diagram of the computational domain of the fish school.

In this section, we discuss the simulation results. The leader was numbered Fish 1, and the followers were numbered Fish 2 and Fish 3. The swimmer was placed in a calculation area of 20 L×12 L, as shown in Figure 8. The Reynolds number was set as Re=1000 and defined as $Re=\rho U_{0}L/\mu$. The movement parameters of the fish leader in each school form were the same, and the parameters were tail amplitude A=0.11 L, body fluctuation period T=250Δt, and wavelength λ=1.0 L. The translation and rotation of the bodies were realized by Equations (20) and (21). The leader, swimming alone under the above exercise parameters, obtained a value of ${CoT}_{s}=0.425$ and an average speed of ${\bar{u}}_{s}/U_{0}=0.284$, where the subscript “s” indicates swimming alone. The fluctuation period T and wavelength λ of the follower fish were the same as those of the leading fish. Only the tail amplitude and phase of the follower fish were changed, and the initial spacing of the follower fish was different from that of the fish leader. The specific parameters are shown in Table 1. The influence of the side-by-side formation, staggered formation, triangle formation, and the different motion parameters of the fish school will be discussed in Section 3.2.1, Section 3.2.2 and Section 3.2.3.

#### 3.2.1. The Side−by−Side Formation

Fish 2 adopted two different phases of φ=0 and φ=1.5π, and there were five initial spacings, as shown in Table 1. Due to repulsion between Fish 1 and Fish 2, none of the cases saw them maintain their initial spacing. In the case of Gy=0.5 L and φ=1.5π, there was a collision between Fish 1 and Fish 2. To explore the influence of the side-by-side formation with respect to the speed and *CoT* of the fish school individual, the speed gain was defined as $(\left(\bar{u}-\bar{u_{s}}\right)∕\bar{u_{s}})\times 100\%$, and the *CoT* gain was defined as $(\left(CoT-{CoT}_{s}\right)∕{CoT}_{s})\times 100\%$.

##### Effect of the Side-by-Side Formation on Swimming Speed

**Figure 9:**
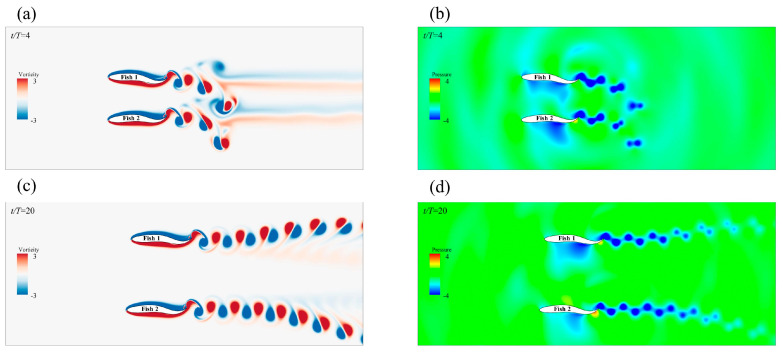
The instantaneous vorticity (**a**,**c**) and instantaneous pressure (**b**,**d**) of the side−by−side formations in the same phase at Gy=0.6 L.

**Figure 10:**
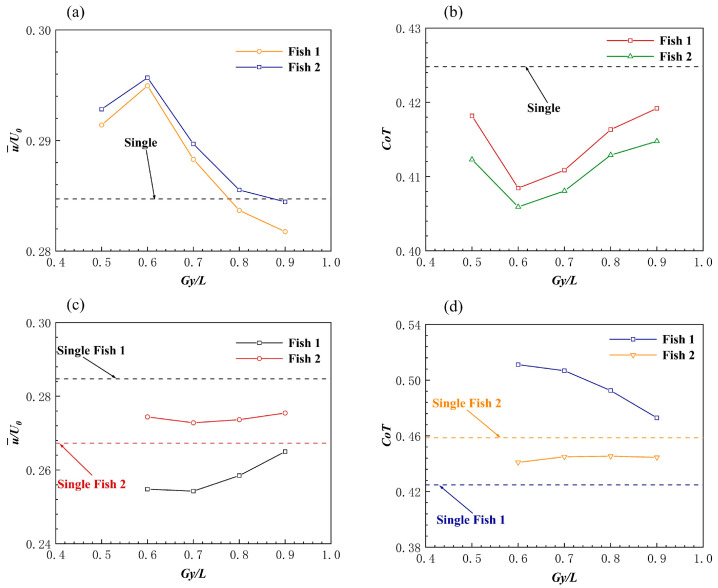
The average forward velocity (**a**,**b**) and the *CoT* (**c**,**d**) of the staggered formations at the follower values of φ=0 and φ=1.5π, respectively.

Since the side-by-side formations did not maintain the initial spacing in all examples, the effect of the propulsion speed of the individuals in the side-by-side formation from the initial separation to the stable spacing will be discussed. Different spacings of side-by-side formations can have speed effects on self-propelled fish [25]. When the Fish 2 motion parameter was φ=0, the side-by-side formation quickly separated, almost within 10 motion cycles, and it resulted in a new stable spacing side-by-side formation. Figure 9a,c show two instantaneous vorticity profiles with an initial spacing of Gy=0.6 L and a Fish 2 phase of φ=0, and the spacing between Fish 1 and Fish 2 changed from dense to sparse. Figure 10a shows the average forward velocity of Fish 1 and Fish 2 as a function of the initial spacing. The forward speed of Fish 1 increased first and then decreased with the change in the initial distance, and the change trend of the forward speed of Fish 2 was the same as that of Fish 1. Therefore, the side-by-side formation in the in-phase did not help the individual forward speed a great deal. The formation divergence, due to the repulsion between Fish 1 and Fish 2, was responsible for this phenomenon. Figure 9b,d show the instantaneous pressure field. At t/T=4, a low pressure area appeared between Fish 1 and Fish 2, and the separation was caused by the unbalanced moment and torque.

**Figure 11:**
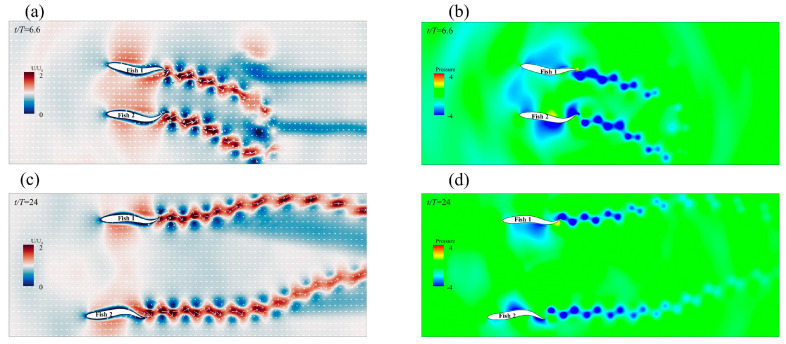
Instantaneous velocity (**a**,**c**) and the pressure (**b**,**d**) of the side−by−side formation when Gy=0.9 L and the follower phase was φ=1.5π.

When the phase of Fish 2 changed to φ=1.5π, Fish 2 approached Fish 1 due to the change in motion parameters, which then resulted in separation again because of the repulsion phenomenon. When φ=1.5π, unlike when φ=0, the structure of the side-by-side formation changed. The flow velocity profiles in the x-direction at two different moments are shown in Figure 11a,c. The side-by-side formation was changed to a staggered formation, where Fish 2 was slightly ahead of Fish 1, and the distance between them in the y-direction was too large. Figure 10c shows the curves of the average propulsion speed of Fish 1 and Fish 2 changing with distance, which occurred when the phase difference was 1.5π. Due to the collision between Fish 1 and Fish 2 at Gy=0.5 L, the average velocity of Gy=0.5 L was not included in the figure. Due to the phase change in Fish 2, the speed of Fish 1 at different Gy was always reduced, and the maximum speed reduction was 10%. Fish 2 slightly increased its forward speed, compared to when swimming alone. To summarize, the side-by-side formation was of little help to the propulsion speed of the self-propelled body; moreover, negative returns occurred. Certain previous studies have also proved this point [42,55].

##### Effect of the Side-by-Side Formation on Energy Efficiency

Figure 10b shows the impact of the different initial distances on the fish school transportation cost (*CoT*) when φ=0. Both Fish 1 and Fish 2 benefitted from it, and they spend less in terms of *CoT* than when swimming alone. Fish 1 showed a maximum *CoT* reduction of 3.8% compared to when swimming alone. The *CoT* change trend of Fish 2 was the same as that of Fish 1, and the energy saving efficiency was at its highest at Gy=0.6 L, whereby the *CoT* was reduced by 4.4%. When the Fish 2 phase was φ=1.5π, the results of the impact of different initial distances on the fish school transportation cost (*CoT*) are shown in Figure 10d. The *CoT* expenditure of the transportation cost of Fish 1 in the side-by-side formation always increased, and the *CoT* expenditure of Fish 2 always decreased. Fish 1 had a *CoT* increase of 20% at Gy=0.6 L, and the energy saving efficiency was also at its lowest. The energy saving efficiency of Fish 2 always improved, and the average reduction in the *CoT* expenditure was about 3.2%.

This is because Fish 2 caused a strong repulsion effect on Fish 1 during exercise, and Fish 1 always increased its power to adjust its body position. The pressure on the bodies of Fish 1 and Fish 2 is shown in Figure 11b,d. At the beginning of the movement, Fish 1 gradually deflected to the right under the action of the pressure difference. This eventually led to the destruction of the side-by-side formation. The *CoT* of Fish 1 was always spent on the deflection process that was caused by the repulsion phenomenon, which was also the worst result for Fish 1; however, for Fish 2, it was the most beneficial result. To summarize, the side-by-side formation was not particularly helpful to the energy saving efficiency of fish swimming; in fact, the energy saving efficiency may have been reduced, which was destructive to the swimming of the fish.

#### 3.2.2. The Staggered Formation

The staggered morphology has been considered beneficial to fish schools and individual hydrodynamics in previous studies. In the case of the staggered formation, the free movement staggered form of the leader and the follower within 50T is stable; in addition, there was no collision between Fish 1 and Fish 2.

##### Effects of the Staggered Formation on Swimming Speed

**Figure 12:**
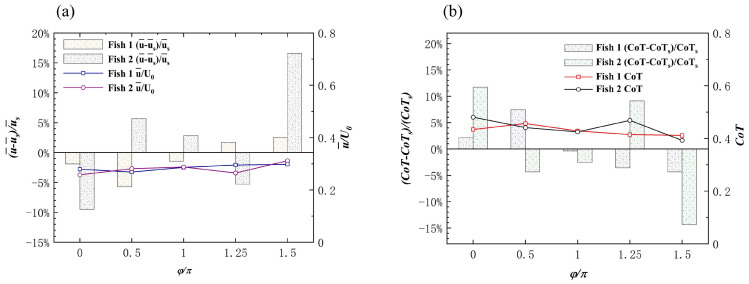
(**a**) Effects of the follower phase changes on the forward velocity of staggered formation individuals. (**b**) The effect of the follower phase change on the *CoT* of the staggered formation individuals. The bar graph indicates the actual speed and *CoT* of the individuals in the fish school, and the histogram indicates the increase in speed and *CoT* when compared to swimming alone.

As shown in Figure 12a, the average speed $\bar{u}/U_{0}$ of the leading fish and the follower fish under different phase differences is given. Here, the phase of the leading fish did not change, but the average speed was $\bar{u}/U_{0}$, as obtained by the influence of the movement of the follower fish in different phases.

**Figure 13:**
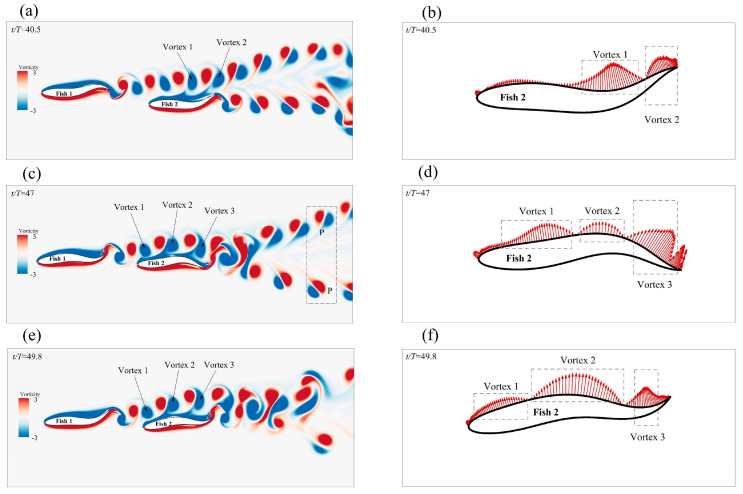
Schematic diagram of the instantaneous vorticity (**a**,**c**,**e**) and the contour and body pressure (**b**,**d**,**f**) of the staggered formation when the follower was φ=1.5π.

Compared with swimming alone, the staggered formation had less of an effect on the forward speed of the leader; in fact, it even reduced the speed. As shown in Figure 12a, Fish 1 was affected by the phase increase in Fish 2, and the speed first decreased and then increased. The speed gain of Fish 2 varied between positive and negative as the phase changed, and the value of φ=1.5π increased by 16% when compared to swimming alone. The velocity gain phenomenon in the staggered form can be explained by the vorticity of the two fish in the flow field. The instantaneous vorticity contours at different moments are shown in Figure 13a,c,e. The motion parameters of the follower Fish 2 were A=0.11 L, λ=1.0 L, and φ=1.5π. At t/T=40.5, it could be observed that the wake vortex generated by the leader acted on the side of the followers’ bodies. The wake vortex generated by the leader had an effect on the flow velocity on the edge of the followers’ bodies as the relative velocity of the fluid was reduced, thus causing the follower to reduce drag when it propelled forward [56]. The formation of a 2P wake structure could be observed in the wake area at t/T=47. The 2P wake structure has always been considered to be the cause of performance enhancement [13].

Figure 13b,d,f give a schematic diagram of the pressure on one side of Fish 2’s body at different times. At t/T=49.8, it was observed that the vortex generated by Fish 1 led to an increase in the pressure on the rear side of Fish 2’s body, and this pressure that was acting on the tail contributed to the generation of the Fish 2 net thrust. At t/T=47, it could be clearly observed that, when the tail of Fish 2 moved from the bottom to the top, the presence of pressure enhanced the effect of the movement. At the same time, because of the existence of the vortex, Fish 2 had a strong suction force; as such, the side of the Fish 2 body was always propelled close to the vortex during movement.

##### Effect of the Staggered Formation on Energy Efficiency

A lower cost of transportation (*CoT*) means a lower energy consumption of the fish moving forward. The impact of the alternation on the leader and the follower will be discussed from the perspective of the changes in energy consumption.

As the phase of Fish 2 changed, the change in *CoT* resulted in a staggered formation, as shown in Figure 12b. As the phase of Fish 2 changed, the *CoT* of Fish 1 showed a trend of first increasing and then decreasing. Fish 1 had the lowest energy saving efficiency when φ=0.5π, and the *CoT* also increased by 7.4%. Fish 2 alternated between positive and negative *CoT* gains as the phase changed, when compared to swimming alone. Fish 2 had the highest energy saving efficiency when φ=1.5π, and the *CoT* was also reduced by 14.3%. For the follower fish, the vortex generated by the leader interacted with the shear layer of the body, which always resulted in the follower extracting energy from the vortex and benefiting from it [57]. This behavior not only increased the speed (as shown in Figure 13a,c), but also meant less effort was required to maintain balance on one side of the body. As shown in Figure 13d,f, the follower, Fish 2, could swim more efficiently because the pressure generated by the passive hydrodynamics was in the same direction as the body’s movement. The reduction in power and increase in speed resulted in a lower *CoT* when the follower moved on the side of the vortex. When regarding the image of the leader, Fish 1, as a passive player, there was not a great deal of benefit to the fish school. It was not difficult to see that, when the phase of the follower changed, its trajectory was also changed, and this phenomenon also determined whether the follower’s body was close to the wake vortex generated by the leader and whether they extracted energy from it.

#### 3.2.3. The Triangle Formation

This section will discuss the hydrodynamics of the fish school when a triangle formation is formed by a leader and two followers. The main changes in the motion parameters of the two followers in the triangle formation were the body fluctuation amplitude A and phase φ. The motion parameters of the two followers were changed at the same time, and the parameters of the leader were fixed, as in the previous two cases.

##### Effects of the Triangle Formation on Swimming Speed

**Figure 14:**
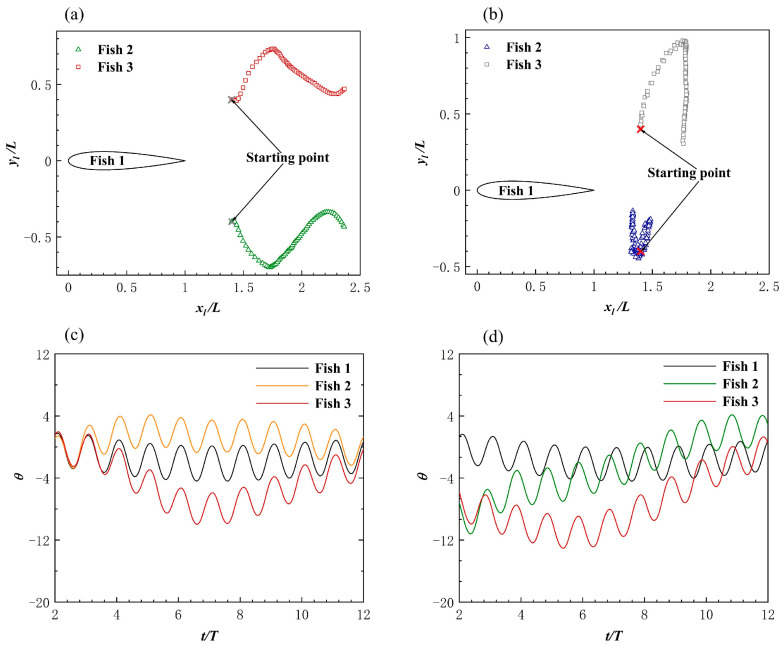
The trajectory of the follower in the leader local coordinate system (**a**) when A=0.105 L and φ=0, and the variation of the deflection angle of the triangle formation within 10 cycles (**c**). The trajectory of the follower in the leader local coordinate system (**b**) when A=0.115 L and φ=1.5π, and the variation of the deflection angle of the triangle formation within 10 cycles (**d**).

When the body fluctuation amplitude A and phase φ of the follower Fish 2 and Fish 3 were changed, their horizontal and vertical distances from the leader changed passively. It is clear that the fish school had different hydrodynamic effects due to the changes in individual spacing. Certain previous studies have proved this point [58]. The trajectory of the followers’ heads in the leader’s local coordinate system, when the follower motion parameters are A=0.105 L and φ=0, is shown in Figure 14a.

**Figure 15:**
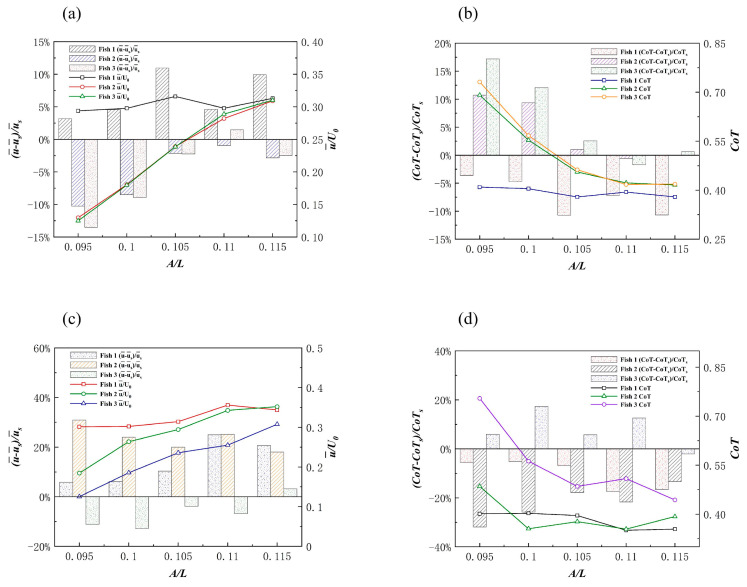
The influence on individual forward speed (**a**) and individual *CoT* (**b**) when the followers and the leader are in the same phase. The influence on individual forward speed (**c**) and individual CoT (**d**) when the followers and the leader are in out of phase. The bar graph indicates the actual speed and *CoT* of individuals in the fish school, and the histogram indicates the increase in speed and *CoT* compared to when swimming alone.

**Figure 16:**
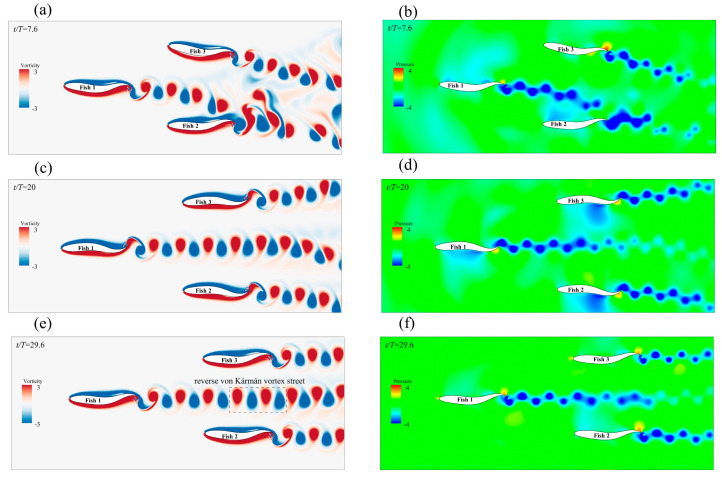
The instantaneous vorticity (**a**,**c**,**e**) contour and instantaneous pressure (**b**,**d**,**f**) field of the triangle formation when the followers and the leader are in the same phase.

The payoff from a triangle formation was always positive for the leader. As shown in Figure 15a, the leader gained speed because the followers’ A value changed. As a result of being affected by the presence of the followers, Fish 1’s speed increased by 10.9%. For the followers, Fish 2 and Fish 3, it appeared that a triangle formation produced the worst speed gain, as it was always negative. With the gradual increase in A, the fish school became denser, and the situation eased. Fish 1’s speed increase was due to the “slapping” motion of the followers behind it, which occurred due to swinging. As shown in Figure 14c, the bodies of Fish 2 and Fish 3 rotated in a larger fashion behind Fish 1 at the beginning of the movement. Certain previous research has shown that this “slapping” action is positive for leaders [59]. Figure 16a,c show the instantaneous vorticity profile of the triangle formation, from the initial movement to the steady state. In the steady state, there was a set of reverse von Kármán vortex streets that were generated by Fish 1 between Fish 2 and Fish 3. Since Fish 2 and Fish 3 had almost advanced in a side-by-side formation, their performance was affected by the combined effect of the reverse Kármán vortex that was generated by the leader and the side-by-side formation; thus, the forward speed was, similarly, always reduced.

As shown in Figure 15c, the velocity increased more for the leader when it was out of phase with the follower. Fish 2’s speed gain was always positive, and Fish 3’s speed gain was always negative. The trajectory of the follower in the local coordinate system of the leader when they were out of phase is shown in Figure 14b, and it can be seen that the followers did not leave the leader. The phase difference between the followers and the leader was the cause of this phenomenon. As shown in Figure 14d, the deflection directions of Fish 2 and Fish 3 were the same. To summarize, the leader, Fish 1, could always obtain an increase in forward speed from the fish school, without making any changes in their motion parameters, and the speed gain brought by the followers from the triangle formation was a double-edged sword.

##### Effects of the Triangle Formation on Energy Efficiency

The followers, Fish 2 and Fish 3, appeared to be a rear-mounted “engine” for the leader, Fish 1, in the triangle formation, thereby providing an increase in speed while improving energy efficiency. Figure 15b shows that the leader and followers were in phase, and that the leader gained energy efficiency gains with the change in the followers’ A. Compared with Fish 1 swimming alone, Fish 1 obtained *CoT* benefits from the triangle formation as the fluctuation amplitude A of Fish 2 and Fish 3 gradually increased. The energy saving efficiency of Fish 1 gradually improved, and the “engines” of Fish 2 and Fish 3 were closer to the root cause of the tail of Fish 1 with an increase in A. It appears as though there was a burden for the followers, Fish 2 and Fish 3. Figure 16b,d,f show the instantaneous pressure field of the triangle formation. The vortex of the leader caused the followers’ original side-by-side low-pressure area to become strengthened, and the followers needed to spend more *CoT* to swim forward.

**Figure 17:**
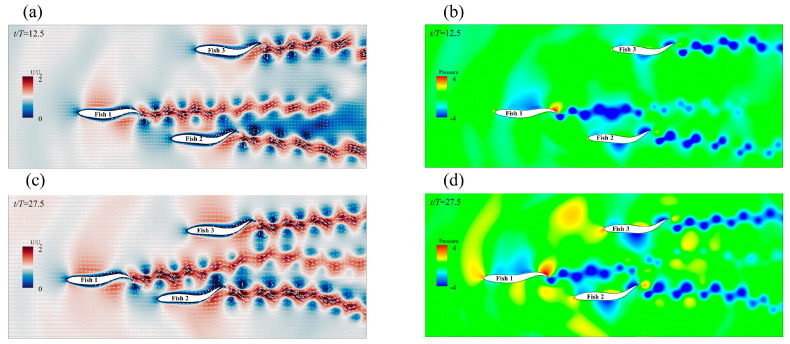
The instantaneous x-direction flow velocity (**a**,**c**) and instantaneous pressure (**b**,**d**) field of the triangle formation when the followers’ φ=1.5π.

As shown in Figure 15d, the *CoT* of Fish 1 always reduced and the energy saving efficiency was higher than that of the same phase. The energy saving efficiency of Fish 1 in a delta formation was higher than that of non-in-phase scenarios, which was as a result of the changed structure of the delta formation. It appeared that the triangle formation, when Fish 2 and Fish 3 were not side-by-side, resulted in a lower *CoT* payout for the individual fish. Figure 17 shows the velocity field of the instantaneous x-direction flow. Due to the presence of Fish 1, the flow velocity in the x-direction on the right side of Fish 2 was slightly enhanced. This enhanced flow velocity led to an enhanced low-pressure area on the right side of Fish 2. Since the direction of the pressure difference was the same as the direction of Fish 2’s movement, it resulted in greater energy efficiency in Fish 2’s movement. To summarize, Fish 1 was always energy efficient in the triangle formation, and the overall energy saving efficiency of the fish school structure was also greatly improved. However, for follower Fish 2, it appeared as though the energy saving efficiency was only improved in the example of φ=1.5π, and for follower Fish 3, it appeared that the triangle formation was the worst in this regard.

### 3.3. Discussion on the Simulation Results

There was a phenomenon of “attraction” and “repulsion” in the side-by-side formation, and this was caused by the pressure field and the non-equilibrium moment of the fish bodies. The negative impact on hydrodynamics caused by the side-by-side formation was due to the low-pressure area between individuals. The reason for this phenomenon was due to the low-pressure area that exists between individuals in side-by-side formation. The low-pressure area produced an “attraction” effect in the initial stage of movement. The individuals always adjusted their body posture to avoid being “attracted”. In this process, the fishes’ movement increased transportation costs. In order to avoid the negative hydrodynamic phenomena caused by side-by-side formations, the initial lateral spacing can be increased to reduce the intensity of the “attraction” effect. For individuals that are in out-of-phase scenarios, the side-by-side formations appeared to receive little help from the school of fish. This is due to the changes in the movement parameters of the fish and the influence of the fluid, which causes the positions of individual fish groups to “separate” rapidly in a short period of time. This phenomenon is observed in Figure 11. In the end, the distance between individuals is too large, thus causing the fish groups to appear to be swimming separately.

Due to the structural specificity of the staggered formation, the possibility of the individuals benefiting from the wake structure generated by the fish school was increased. This possibility could be related to the phase difference between the individuals and the influence of passive hydrodynamics, which determine whether the followers’ trajectory can be close to the vortex that is generated by the leader. The main performance parameter that the follower obtains energy from is the vortex structure, which reduces forward resistance. For the leader, the staggered formation structure is sparse due to changes in motion parameters of the follower, which weakens the influence of hydrodynamics.

In the triangle formation, it was found that the vortex trajectories generated by the leader existed among the followers. These vortices provided energy for the followers in the triangle formation to move forward, and reduced the forward resistance on the side of the followers. The existence of followers added two “wings” to the leader, thereby changing the fluid environment behind the leader. This generated additional momentum in the same direction as the leader’s movement, thus increasing the leader’s forward speed. The current results are supported by previous research [25].

To sum up, in order to avoid the negative effects caused by side-by-side formation, the lateral spacing can be increased. The fish body should be close to the vortex during movement to obtain energy from it. A stable structure of the fish school should be maintained. The current work provides suggestions for future bionic fish to gain energy savings and speed improvements through fish schools.

## 4. Conclusions

The purpose of this study was to understand the hydrodynamic effects of different swimming parameters and spacings between individual fish on fish schools. Through numerical simulation, and under the initial conditions of a natural flow, the hydrodynamic performance of a 3-DOF self-propelled fish in three formations, namely side-by-side, staggered, and triangle, was analyzed.

In terms of hydrodynamic performance, this study shows that, since the individual degrees of freedom in the fish school were opened—under the influence of the pressure field and torque—the “attraction” and “repulsion” phenomena between individuals occur, and these affect the stability of the fish school structure. A stable fish school structure is the key for individuals to benefit from the swarming effect, which is mainly reflected in energy and speed. The existence of vortices changes the shear layer of the fish bodies, thus causing resistance during movement to decrease and the speed to increase.

The existence of the pressure field causes the formation to often develop from high density to low density, eventually forming a new stable spacing. The wake vortex produced by the leader is a slender low-pressure area, and the follower uses the low-pressure area to act on one side of the pressure difference to strengthen the body’s effort saving movements. The followers act as an additional source of power, and the presence of the followers results in a stronger pressure zone behind the leader, thus resulting in a stronger hydrodynamic performance.

This study contributes to the development of bionic engineering, as well as providing a theoretical basis. However, this study has limitations. The fish school structure was easily damaged by hydrodynamic forces, such as collision or separation between individuals. In future work, we will consider introducing a deep reinforcement learning control strategy to ensure the fish school formation.

## Figures and Tables

**Table 1:** The different fish school cohorts and phase differences considered in this work.

Formation Type	Geometric Figure	Gx (L)	Gy (L)	φ (π)	A (L)
Side-by-side	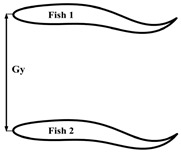	0	0.5, 0.6, 0.7, 0.8, 0.9	0, 1.5	0.11
Interlace	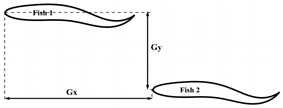	1.6	0.6	0, 0.5, 1.0, 1.25, 1.5	0.11
Triangle	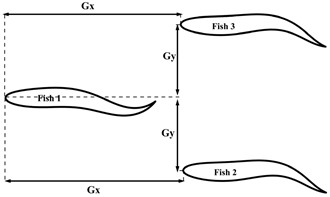	1.4	0.4	0, 1.5	0.095, 0.10, 0.105, 0.11, 0.115

## Data Availability

The data supporting this study are presented in the article.
